# Integrating Patient-Generated Health Data Throughout the Total Product Life Cycle of Medical Devices

**DOI:** 10.1007/s43441-023-00505-5

**Published:** 2023-06-09

**Authors:** Christina M. Webber, Anne Riberdy Hammer, Anindita Saha, Danica Marinac-Dabic, Daniel A. Caños, Michelle E. Tarver

**Affiliations:** Center for Devices and Radiological Health, U.S. Food and Drug Administration, 10903 New Hampshire Ave, Silver Spring, MD 20993 USA

**Keywords:** Patient-generated health data, Medical devices, Registries, Patient-driven registries, Real-world data

## Abstract

Nearly ubiquitous use of personal electronics, wearable sensors, and other types of digital health technologies, along with wireless connectivity, makes the capture of health data directly from an individual easier, enabling the use of patient-generated health data (PGHD) as a potential bridge between a patient’s home and the healthcare system. This type of real-world data may be a completely new type of information, or it may be a more frequent collection of traditional information over longer time periods to form a longitudinal view of a patient’s health status that can inform decision-making in clinical, medical product regulatory, and coverage and reimbursement settings. The U.S. Food and Drug Administration’s Center for Devices and Radiological Health (CDRH) has been exploring and advancing the collection and use of PGHD since 2016, hosting a public meeting on the topic in May 2021. This manuscript presents highlights from various discussions at this meeting including those on the importance of stakeholder engagement, characteristics of high data quality, and PGHD in practice in patient-driven registries, as well as a look forward to some of the opportunities in the field.

## Introduction

Insights into patients’ daily lived experiences are often collected using patient-reported outcome (PRO) measures, which can include questions relating to symptoms and functional abilities summarized over a specified period of time. However, the collection and use of these and other types of patient-generated health data (PGHD) offers the potential opportunity to assess aspects of their experience continuously. Ways to measure PGHD have accelerated in the digital age. Nearly ubiquitous use of personal electronics, wearable sensors, and other types of digital health technologies, along with wireless connectivity, makes the capture of health data directly from an individual easier, enabling the use of PGHD as a potential bridge between the patient’s home and the healthcare system. The Office of the National Coordinator for Health Information Technology defines PGHD as data “created, recorded, or gathered by or from patients…to help address a health concern” [1]. A distinguishing feature of PGHD is that patients, not providers, are the ones responsible for capturing and sharing these data. Sources of PGHD include wearable digital health technologies (e.g., pedometer, blood glucose monitor), patient-driven registries, social media, and mobile applications (e.g., electronic PRO instruments).

Throughout the healthcare ecosystem, healthcare providers, regulators, payors, and others may use PGHD to help form a more comprehensive view of an individual’s health status, expanding what is typically gleaned from a traditional clinical or research visit. This type of real-world data may be a completely new type of information, or it may be a more frequent collection of traditional information over longer time periods to form a longitudinal view of a patient’s health status. It is possible that this data could support regulatory decision-making for medical products or be used in other decision-making scenarios [2, 3].

The potential impact PGHD can have on healthcare is great and still to be fully realized. The U.S. Food and Drug Administration’s Center for Devices and Radiological Health (CDRH) has been exploring and advancing the collection and use of PGHD, beginning formalized efforts in July 2016 with the release of a draft guidance on real-world data, which was issued as final guidance in August 2017. Subsequent efforts by CDRH at the 2018 Patient Engagement Advisory Committee meeting sought to better understand how patients viewed the opportunities and challenges associated with the use of PGHD in post-market surveillance [4]. This meeting raised additional questions about the ways PGHD could be used across the entire total product life cycle of a medical device. To further explore these questions, CDRH held a public meeting in May 2021, bringing together stakeholders from across the healthcare ecosystem [5]. Robust discussions focused on the need for stakeholder engagement and high data quality to facilitate the use of PGHD across the medical device total product life cycle, including in clinical trials, clinical care, coverage and reimbursement decisions, and health surveillance. This manuscript presents highlights from those discussions, synthesizes views provided by stakeholders present at the meeting, as well as provides a look forward to some of the opportunities in the field as discussed at the public meeting.

## Engaging of Stakeholders

Involving multiple stakeholders to design, produce, collate, and analyze data can result in rich data sets. When establishing a patient-driven registry or other platform to host PGHD, it is important to engage a variety of stakeholders with different expertise and perspectives to clearly define the purpose of collecting PGHD and the role each stakeholder plays in this collection. For example, patients and healthcare professionals can help identify core concepts, including relevant clinical outcomes, and how they can be incorporated as standardized data elements. Healthcare professionals may also be able to contribute clinical data and metadata to complement PGHD in a database or registry platform. Additionally, informaticians, epidemiologists, and statisticians can lead vocabulary coordination among stakeholders, along with metadata development, data element specification, and data element modeling which are all critical elements for collecting robust data. To build a platform that fulfills stakeholder needs, process engineers and health information technology vendors can work together and focus on integrating data capture that is interoperable and least burdensome to patients. Overall, collaborative efforts among a variety of stakeholders can result in the development of a robust platform to collect, collate, and analyze PGHD.

Focusing specifically on engagement of the patient community, data that comprehensively represents the patient experience could be used to inform decision-making in clinical, medical product regulatory and/or coverage and reimbursement settings. Central to this engagement is building and fostering patient trust around who is collecting the data, how data will be collected and stored, its intended use, who will have data access, and whether use of the data will ultimately benefit the communities that are contributing. Digital data collection has emphasized the importance of intentionally cultivating trusted relationships with patients, patient groups, and community organizations to further advance the generation of clinically meaningful data. Sustained and authentic relationships between the patient groups that are generating and providing data and data users help to provide reliable and valid information leading to supportable inferences. Ways in which trust can be fostered include ensuring the data sharing is mutually beneficial, appropriately compensating individuals for their time and efforts, sharing findings with the communities that contribute the data, and pursuing sustained relationships rather than transactional ones [6].

## Ensuring Quality Data

High-quality PGHD can inform sound decisions and relies on various elements that may be prioritized differently by different stakeholders. Some may place more emphasis on a robust lexicon of measures that need to be captured, while others may focus specifically on the technology platforms that are used to collect, curate, validate, and aggregate data. By beginning with a quality-by-design mindset, stakeholders can focus on building a system where collected PGHD is able to withstand audit and provide relevant information for the intended purpose. Existing clinical registries evaluating a specific condition or product of interest can be synthesized and leveraged when they are developed with consistent, harmonized definitions of relevant core clinical concepts, known as common data elements. These harmonized definitions of common data elements improve data interoperability and allow for seamless data flow from the patient to the platform and then to researchers or healthcare providers. One standardized element that is helpful to include when describing medical devices is the Unique Device Identifier (UDI). The UDI is a unique numerical or alphanumerical code that identifies the product labeler, specific version or model of a device, lot or batch number, serial number, expiration date, along with other information [7]. Inclusion of this standardized information as a common data element, when appropriate, can be useful for device tracking and can facilitate medical device surveillance efforts. Applying thoughtful data cleaning and quality checks, including anticipating oddities, discrepancies, and/or logic inconsistencies between patient reports and clinical records, can help ensure PGHD meets the standards and expectations of stakeholders. Quality checks can also help identify and address “bots” (i.e., automated software) or fake patients that may skew data. Additionally, actively monitoring data quality and listening to patient feedback can aid in identifying barriers to obtaining high quality data. Prior to the collection of PGHD via a patient-driven registry or other method, it is important for stakeholders to thoughtfully consider data stewardship, including the governance, management, and ownership of the data, to ensure the integrity of the data collected and that the data collected fulfills its intended purpose and patient privacy is maintained.

## Stewarding Data Responsibly

Responsible data stewardship includes a focus on governance and management of data. Strong and sustainable governance occurs when there is oversight and a framework for patient agency of their data and access to PGHD through a platform. Proactively establishing a secure process for login, data entry, assessment of data integrity and completeness, and third-party data access sets the platform up for success. Further, if data is synthesized from multiple sources, governance could also address any privacy and/or cybersecurity expectations and considerations. Strong partnerships among patients, healthcare providers, healthcare systems, the life sciences industry, payors, government, academia, and other stakeholders can help outline and establish data governance requirements for a given platform.

The ethical management of PGHD, which may include Personal Identifiable Information (PII), is built upon the trust of those who provide the data. Ethics experts, privacy experts, and patients being at the table and engaged prior to and continuously throughout data collection can help ensure that the collected data serves all specified purposes in a secure fashion. Transparency surrounding the methods and technologies employed to collect, synthesize, store, and transmit PGHD also plays a crucial role in cultivating patient trust and ensuring the integrity of the data. The ability for patients to opt in or out of the PGHD collection platform and associated research studies may further foster trust with patients. For data access, burdens related to cost and accessibility can be minimized to enable the patients to easily share or grant access to complementary sources of data.

## Collecting Data

Advanced and detailed planning can facilitate the sustainable collection of PGHD. Establishing data use principles, with special focus on maintaining patient privacy when PGHD is accessed by third parties like researchers or healthcare providers, could be an important part of this planning. Easy-to-use and secure technical platforms encourage sustained patient participation. In particular, user accessibility testing prior to platform deployment can help evaluate the ease of providing consent, logging into the system, and inputting data to the platform consistently.

Engaging patients throughout the data collection process can help mitigate potential missing data and maximize the value of collected data. It is important to consider how data collection could be integrated into daily life in the least burdensome way for patients. The ability to skip questions may help patients maximize their contributions but can lead to sparce data if the entry format is too unstructured or the skip patterns are not aligned with the research objectives. Other critical considerations to help ensure data collected is of high quality and relevance are a patient’s comfort level in providing the requested data, as well as the amount and level of detail that is realistic for a patient to provide. Ensuring that data collection, including login and data entry, are patient-centered can help minimize the amount of missing data and ensure that the data collected is meaningful to not only researchers and healthcare providers, but patients as well.

## PGHD in Practice: Patient-Driven Registries

Patient-driven registries can serve as a platform to collect and host PGHD from a patient population who has been treated with a type of medical product, are impacted by a specific disease or condition, or have received a common therapy or medical procedure. Registries potentially have a global reach and often offer a longitudinal view of a patient’s health status. To complement PGHD from sources like electronic PRO instruments and wearable digital health technologies, many patient-driven registries also leverage data from electronic health records, health insurance claims, laboratory data, and genetic testing. Patient-driven registries are distinct from clinical registries because patients or patient advocacy groups control data generation, collection, and management. Furthermore, the structure of patient-driven registries can allow for the collection of data that is meaningful to patients, providing a more complete view of the impact a medical product, disease or condition, or treatment or medical procedure on the patient. Patient-driven registries may also connect patients to relevant research and clinical communities so that they may contribute to the advancement of knowledge, and possibly identify care options. Often, access to patient-driven registries is controlled to protect the security of patient data and researchers must follow specific protocols to request access to the registry data.

To explore how PGHD is being collected and harnessed, representatives from four patient-driven registries shared their experiences during the public meeting. The Fox Insight registry, established by The Michael J. Fox Foundation, enrolls both patients with Parkinson’s disease to serve as participants in clinical trials as well as historical controls [8]. Its broad eligibility criteria and online nature help to avoid typical barriers to research for this patient population, including mobility limitations, transportation challenges, and access to medical and research institutions. CreakyJoints, the Global Healthy Living Foundation’s patient community focused on arthritis, created the ArthritisPower patient-driven registry for patients with rheumatic and musculoskeletal conditions like rheumatoid arthritis and spondyloarthropathies [9]. This research-focused registry shares relevant research with patients and is tailored to optimize patient engagement and adherence to the study protocol. Data is collected actively (e.g., when patients complete an electronic PRO instrument), and passively (e.g., when a patient links their wearable digital health technology to the platform). The National Organization for Rare Disorders recognized the unique needs of populations with rare diseases and built the customizable IAMRARE Registry [10]. Patients own their data and are in control of the access to their data. The Foundation for Fighting Blindness also recognized the needs of patients, specifically those with inherited retinal diseases, and established the My Retina Tracker patient-driven registry [11]. The platform design has been tailored so that patients with low vision can still access the registry which links them to relevant information about their disease, as well as research opportunities in which they may be interested.

Other patient groups can draw upon the experiences and challenges of establishing and managing these patient-driven registries. Continued active engagement of patients in a registry can be difficult, but online registries can help facilitate that by meeting an individual where they are when there is a need for data collection. Additionally, to encourage sustained participation, it is important to consider patients’ motivation to share their data and any ways to ease the potential burden on patients. As with all aspects of healthcare, concerted efforts to include underrepresented populations in registries may help to limit the propagation of healthcare disparities.

## What’s Next?

Decision-makers, including regulators, payors, healthcare providers, and patients, have exponentially increasing amounts and types of data available to them to help inform their decisions. While there is not a “one size fits all” answer to how PGHD can be considered in different types of decision-making, advancements to improve the transparency of PGHD to all stakeholders may help integrate this type of data in more decisional frameworks. Whether it is clinical trials incorporating elements of PGHD or expanding real-world surveillance efforts with PGHD tools, determining how to analyze and resolve information collected during clinical visits with information collected in the home will depend on the needs of the stakeholder and the associated research question. Furthermore, developing novel analysis and visualization techniques or modifying established ones may help organize the vast amounts of PGHD that can now be collected and enable its understanding by researchers, patients, and healthcare providers, among other stakeholders. In addition to the quantity of data now available, the novel tactics to impute or improve the quality of PGHD is an area for future work.

Integrating PGHD, like data from a wearable digital health technology or an electronic PRO instrument, into clinical practice and decision-making offers the potential to improve quality of care, facilitate patient-provider discussions, and help bring to light health-related information not necessarily known before. However, access and usage disparities exist and threaten this improvement in care quality. While access to digital health technologies and the connectivity required to successfully collect PGHD is expanding, significant gaps in access remain and it is important to avoid exacerbating existing disparities, especially in underserved and underrepresented populations. Alongside assurance of equitable access to the infrastructure required to successfully collect and use PGHD, efforts to build a transparent system as well as cultivate trust are critical to success. Future advancements in the technology, ecosystem infrastructure, and interoperability can help support a seamless exchange of PGHD between patients, providers, and researchers in a responsible manner.

Collaboration among patients, researchers, healthcare providers, electronic health record manufacturers, regulators, payors, health technology assessment bodies, and others offers the potential for efficient and effective advancement of the responsible collection, management, and use of PGHD. Through public–private partnerships, collaborative communities, and other collaborative approaches, FDA encourages all stakeholders to bring their unique perspectives and skills to the table to help advance the collection and use of PGHD throughout the healthcare ecosystem [12–15].

## Conclusions

Patient-generated health data can provide rich types of evidence to be considered in decision-making, as discussed in the May 2021 CDRH public meeting focused on PGHD. Thoughtful efforts when engaging stakeholders to develop platforms that facilitate the collection and use of PGHD can help improve the utility of the data. Moreover, collaborative efforts to advance the impact of PGHD on healthcare can be augmented by involving stakeholders not typically involved in medical product development. Patient-driven registries are already demonstrating the benefits of using PGHD in a responsible manner to collect and harness data directly from patients to help improve care and assess medical products. CDRH remains actively engaged in efforts to explore and expand the use of PGHD in the healthcare ecosystem.

## Data Availability

Meeting materials, including the transcript, used when drafting this manuscript can be found at https://www.fda.gov/medical-devices/workshops-conferences-medical-devices/virtual-public-meeting-patient-generated-health-data-throughout-total-product-life-cycle-medical.

## Declarations

### Disclaimer

This review reflects the views of the authors and should not be construed to represent the views or policies of FDA.
